# A subgroup of pleural mesothelioma expresses ALK protein and may be targetable by combined rapamycin and crizotinib therapy

**DOI:** 10.18632/oncotarget.25111

**Published:** 2018-04-17

**Authors:** Dina Mönch, Sabine Bode-Erdmann, Jörg Kalla, Jörn Sträter, Carsten Schwänen, Roger Falkenstern-Ge, Siegfried Klumpp, Godehard Friedel, German Ott, Claudia Kalla

**Affiliations:** ^1^ Dr. Margarete Fischer-Bosch Institute of Clinical Pharmacology, 70376 Stuttgart, Germany; ^2^ Department of Clinical Pathology, Robert-Bosch-Krankenhaus, 70376 Stuttgart, Germany; ^3^ University of Tübingen, 72074 Tübingen, Germany; ^4^ Institute of Pathology, Schwarzwald-Baar-Klinikum, 78052 Villingen-Schwenningen, Germany; ^5^ Institute of Pathology, 73730 Esslingen, Germany; ^6^ Clinic of Internal Medicine, Oncology/Hematology, Gastroenterology and Infectiology, Klinikum Esslingen, 73730 Esslingen, Germany; ^7^ Center for Pulmonology and Thoracic Surgery, Klinik Schillerhöhe, 70839 Stuttgart-Gerlingen, Germany; ^8^ Hospital Pharmacy, Robert-Bosch-Krankenhaus, 70376 Stuttgart, Germany

**Keywords:** pleural mesothelioma, ALK, crizotinib, rapamycin, combination therapy

## Abstract

Malignant pleural mesothelioma (MPM) is a neoplasm with inferior prognosis and notorious chemotherapeutic resistance. Targeting aberrantly overexpressed kinases to cure MPM is a promising therapeutic strategy. Here, we examined ALK, MET and mTOR as potential therapeutic targets and determined the combinatorial efficacy of ALK and mTOR targeting on tumor cell growth *in vivo*.

First, *ALK* overexpression, rearrangement and mutation were studied in primary MPM by qRT-PCR, FISH, immunohistochemistry and sequence analysis; *mTOR* and *MET* expression by qRT-PCR and immunohistochemistry. Overexpression of full-length *ALK* transcripts was observed in 25 (19.5%) of 128 primary MPM, of which ten expressed ALK protein. *ALK* overexpression was not associated with gene rearrangement, amplification or kinase-domain mutation. mTOR protein was detected in 28.7% MPM, co-expressed with ALK or MET in 5% and 15% MPM, respectively. The ALK/MET inhibitor crizotinib enhanced the anti-tumor effect of the mTOR-inhibitor rapamycin in a patient-derived MPM xenograft with co-activated ALK/mTOR: combined therapy achieved tumor shrinkage in 4/5 tumors and growth stagnation in one tumor. Treatment effects on proliferation, apoptosis, autophagy and pathway signaling were assessed using Ki-67 immunohistochemistry, TUNEL assay, LC3B immunofluorescence, and immunoblotting. Co-treatment significantly suppressed cell proliferation and induced autophagy and caspase-independent, necrotic cell death. Rapamycin/crizotinib simultaneously inhibited mTORC1 (evidenced by S6 kinase and RPS6 dephosphorylation) and ALK signaling (ALK, AKT, STAT3 dephosphorylation), and crizotinib suppressed the adverse AKT activation induced by rapamycin.

In conclusion, co-treatment with rapamycin and crizotinib is effective in suppressing MPM tumor growth and should be further explored as a therapeutic alternative in mesothelioma.

## INTRODUCTION

Malignant pleural mesothelioma (MPM) is an aggressive cancer originating in the pleura. MPM is associated with poor prognosis: less than 5% of patients survive more than five years [1]. Surgery combined with radio-chemotherapy has shown benefit in patients presenting with early-stage disease [2], but most MPM are in advanced stage and show multi-focal growth at the time of diagnosis. The current treatment standard for advanced MPM - chemotherapy with cisplatin and pemetrexed or raltitrexed - is effective only in 25-30% of patients, and median survival is 12 months [3]. There is no approved salvage regimen after failure of first-line chemotherapy. Therefore, new treatment approaches are urgently needed. New drugs targeting aberrantly activated signaling molecules may be candidate therapeutics in MPM.

**Figure 1:**
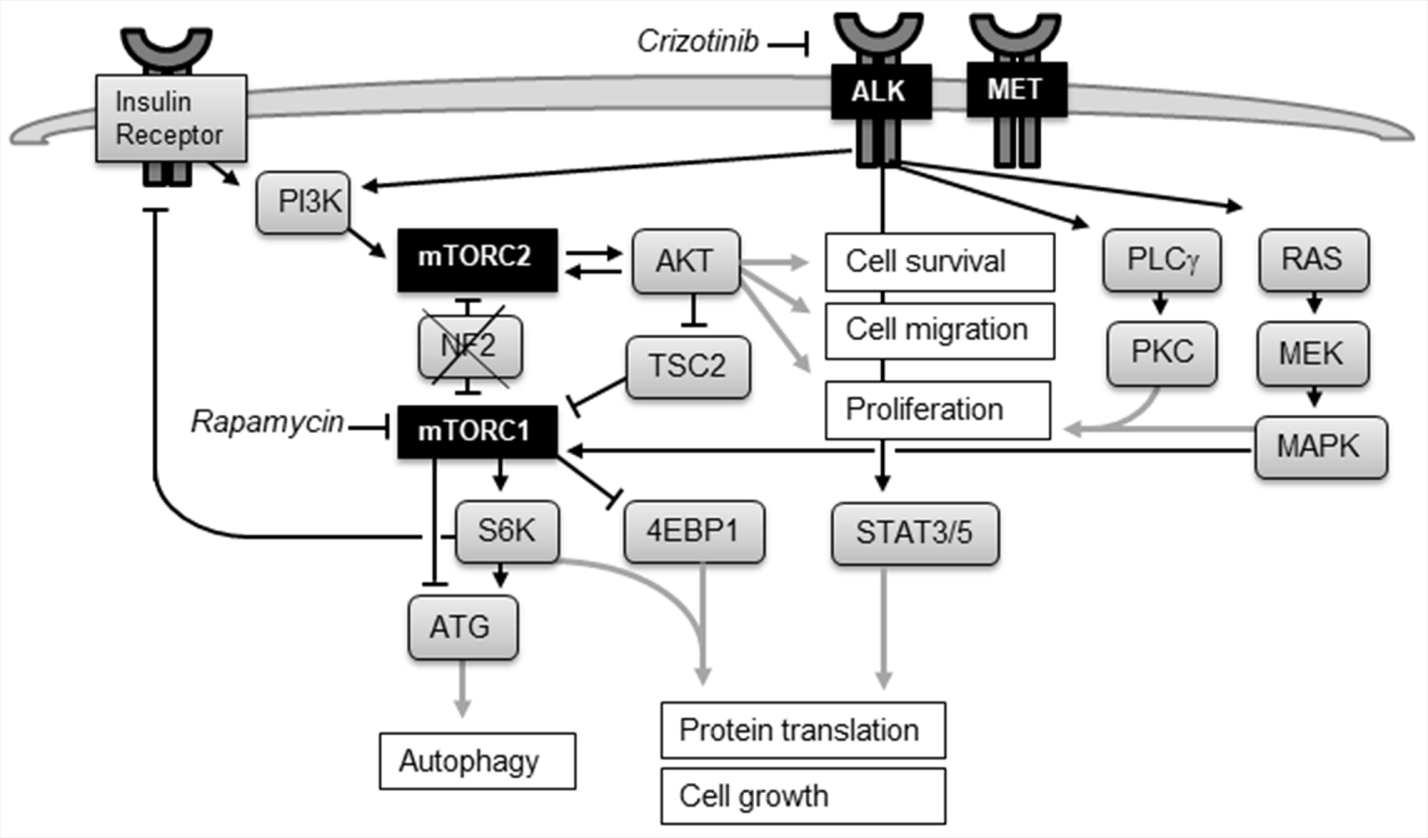
The mTOR and ALK signaling network Activation of tyrosine kinase ALK through ligand binding stimulates downstream signaling through the PI3K/AKT, STAT3, and RAS/MAPK pathways. Deletion of NF2 leads in 40-50% of MPM to aberrant activation of mTOR. mTORC1 promotes protein synthesis through phosphorylation of S6K and 4EBP1 and inhibits autophagy. mTORC2 functions as an effector of PI3K signaling and controls proliferation and survival primarily by activating AKT. Positive feedback loop: partial activation of AKT promotes activation of mTORC2, which in turn phosphorylates and fully activates AKT. Negative feedback loop: mTORC1 suppresses mTORC2 activation through the inhibition of insulin/PI3K signaling. Inhibiting mTORC1 releases the negative feedback and increases AKT activation.

Receptor tyrosine kinases (RTKs) play a crucial role in tumor growth, transducing extracellular signals that regulate cellular proliferation and survival, in particular activating PI3K/AKT and RAS/MEK/MAPK signaling and STAT regulated transcription (Figure 1). The stringent control of RTKs is often abrogated in tumor cells: gene mutations, amplifications, overexpression and gene fusions result in constitutive activation. Specific tyrosine kinase inhibitors have shown remarkable clinical response in patients with tumors characterized by RTK activation, such as gefitinib and erlotinib in EGFR-mutated [4] and crizotinib in ALK- or ROS1-rearranged lung cancer [5, 6]. Crizotinib (PF02341066) is a potent, orally bioavailable, well tolerated, small molecule inhibitor of ALK, ROS1 and MET. It is approved for treatment of ALK- or ROS1-rearranged lung cancer and undergoing clinical trials in patients with other advanced solid tumors.

Various studies have reported on the expression and activation of RTKs in mesothelioma, including EGFR, MET, IGFR, FGFR1, and VEGFR [7–9]. Specific inhibition of VEGFR, FGFR1, MET and/or EGFR suppressed mesothelioma cell growth *in vitro* [7–12]. However, clinical studies with RTK inhibitors as single agents were disappointing. VEGFR inhibition, e.g. had minimal activity in MPM and was poorly tolerated [13, 14], and single-agent EGFR or MET inhibition was clinically ineffective in MPM patients, despite high expression of EGFR [15, 16] and MET (NCT01861301).

Deletion of the tumor suppressor NF2 seen in 40-50% of MPM leads to aberrant activation of the serine/threonine protein kinase mTOR. mTOR coordinates cell growth by regulating protein, lipid and nucleotide synthesis, cell proliferation, survival, and autophagy [17, 18]. mTOR forms the catalytic subunit of two distinct protein complexes, mTOR Complex 1 (mTORC1) and 2 (mTORC2). mTORC1 functions as a downstream effector for the oncogenic PI3K/AKT and RAS/MEK/MAPK pathway and regulates cell size and protein synthesis through its substrates S6K and 4EBP1. The mTORC2 complex regulates cell proliferation and survival through phosphorylation of AKT (Figure 1).

Inhibition of mTOR by rapamycin or its derivatives (sirolimus, temsirolimus, everolimus) suppressed mesothelioma cell growth in pre-clinical models [17, 19, 20], but was not effective in clinical trials: everolimus showed no therapeutic benefit in unselected MPM patients [21] and a small group of patients selected for NF2 deletion (NCT01024946). These disappointing results are most probably due to adverse AKT activation: Inhibiting mTORC1 releases the negative feedback on PI3K/AKT signaling and increases AKT activation (Figure 1), which may promote cell survival and prevent apoptosis [22, 23]. Moreover, mTORC1 inhibition induces autophagy, helping to maintain cancer cell survival [18].

We postulated, therefore, that co-targeting of mTOR and RTK signaling pathways may result in greater therapeutic benefit via simultaneous inhibition of mTORC1, RAS/MEK/MAPK and STAT signaling and simultaneous suppression of rapamycin-induced AKT activation. Consequently, we have elucidated the cellular basis of the combinatorial therapeutic potential of rapamycin and crizotinib in MPM. We performed a screen for aberrantly expressed crizotinib targets in a large panel of MPM tumors and have found ALK and mTOR as well as MET and mTOR co-expression in a subgroup of MPM. We also found that the combined use of rapamycin and crizotinib was more effective than rapamycin as single-agent in suppressing tumor growth in a patient-derived mesothelioma graft with co-activated ALK and mTOR pathways.

## RESULTS

### ALK/MET and mTOR kinases are co-expressed in a subset of primary mesotheliomas

To assess the frequency of co-activation in primary mesotheliomas, we examined the co-expression of *mTOR* and *ALK*, *ROS1*, and *MET* at both mRNA and protein levels in tumor samples by qRT-PCR and IHC, respectively.

**Figure 2:**
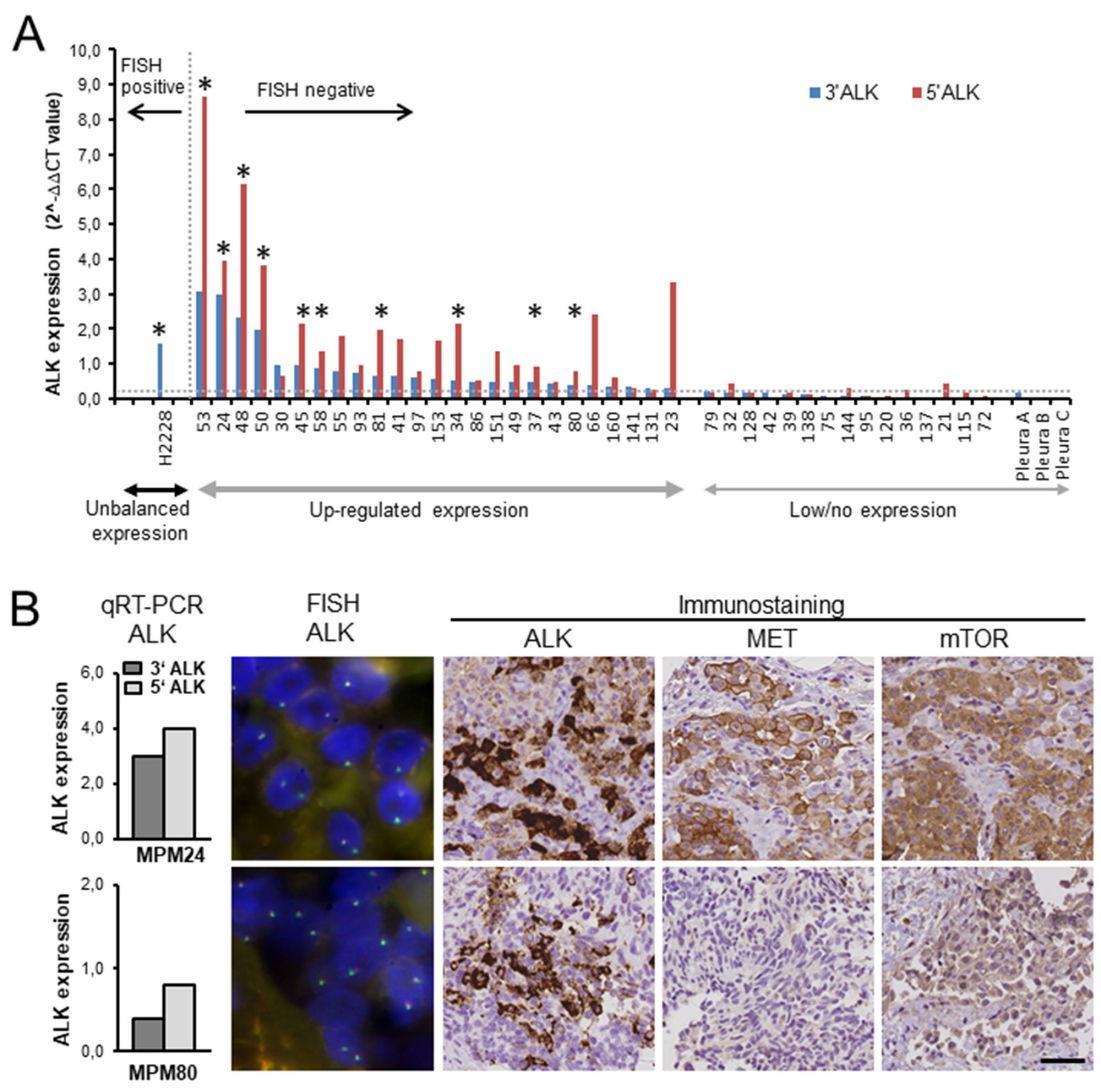
Expression of ALK, MET and mTOR in mesothelioma tumor tissue samples **(A)** qRT-PCR analysis of *ALK* was performed relative to the *PGK1* housekeeping gene. The horizontal dashed line indicates the cut-off value for altered *ALK* expression in ALK positive lung cancer (0.3); [24] blue bars, *ALK* 3‘ portion; red bars, *ALK* 5‘ portion. The asterisks point to tumors expressing ALK protein. Lung cancer cell line H2228 served as positive control showing an unbalanced expression of the *ALK* 3‘ portion due to *EML4-ALK* translocation. **(B)** Analysis of ALK, MET and mTOR expression in MPM24 and MPM80. Upregulated, balanced expression of *ALK* transcripts was independent of translocation, as confirmed by *ALK* break-apart FISH. Scale bar, 200μm.

We used recently developed qRT-PCR assays that reliably detect (1) *ALK* and *ROS1* translocations by recognizing unbalanced expression of the *ALK* and *ROS1* 3’ parts encoding the kinase domain, while the 5’ parts remain unexpressed, and (2) upregulated, balanced *ALK* and *ROS1* gene expression (Figure 2A) [24, 25]. *ALK* qRT-PCR was applied to 128 mesotheliomas and five normal pleura specimens. Unbalanced *ALK* transcript expression indicative of a gene rearrangement was not observed. Instead, 25 (19.5%) tumors showed upregulated balanced expression of *ALK* transcripts, while *ALK* was not expressed in normal pleura. ALK protein was detected by IHC in ten of the 25 samples with upregulated *ALK* transcript expression (Figure 2A, 2B). All ALK protein expressing tumors were confirmed to be negative for genomic *ALK* rearrangements and gene amplifications by FISH (*ALK* copy number: 1.30-1.68; Figure 2B). Because up-regulated *ALK* expression is frequently associated with mutations in the kinase domain in neuroblastoma [26], we examined *ALK* exons 20-29 for mutations in all cases with ALK protein expression. However, activating mutations in the ALK kinase domain were not found. As for *ROS1, MET* or *mTOR* genes, qRT-PCR analysis showed that *ROS1* was not expressed in 126 MPM analyzed. Expression of *MET* or *MTOR* was significantly higher in tumor tissues than in normal pleura in 66/92 (71.7%) and 46/95 (48.4%) MPM, respectively. Immunostaining of ALK, MET and mTOR on tissue microarrays was interpretable in 101 mesotheliomas (Table 1). In this cohort, 10 cases (9.9%) were ALK positive. MET staining was detected in 83 of 101 tumors, of which 46 (45.5%) were moderately to strongly positive. Eighty-five mesotheliomas were immunoreactive for mTOR, of which 29 (28.7%) showed moderate to strong reactivity. Altogether, high-level ALK and mTOR or MET and mTOR co-expression - the basis for kinase co-activation – was observed in 5% and 15% MPM, respectively (Table 1, Figure 2B).

**Table 1:** Expression of mTOR, ALK and MET in mesothelioma tumor tissue samples

	n	ALK positive^1^ MET positive^1^	ALK positive^1^ MET negative^2^	ALK negative^2^ MET positive^1^	ALK negative^2^ MET negative^2^
**mTOR positive**^**1**^	29	2	3	13	11
**mTOR negative**^**2**^	72	4	1	27	40
Total	101	6	4	40	51

^1^Positive refers to moderate or strong IHC staining in at least 10% tumor cells.

^2^Negative refers to weak or no IHC staining in the tumor cells.

Correlation with clinicopathologial parameters revealed that age, sex, asbestos exposition, initial presentation with metastasis, and histological growth pattern (epithelioid, sarcomatoid, biphasic) were not significantly associated with ALK, MET or mTOR protein expression (Supplementary Table 1). In addition, overall survival did not differ between kinase-positive and kinase-negative MPM.

### Combined therapy with rapamycin and crizotinib abrogates MPM growth in a xenograft model

**Figure 3:**
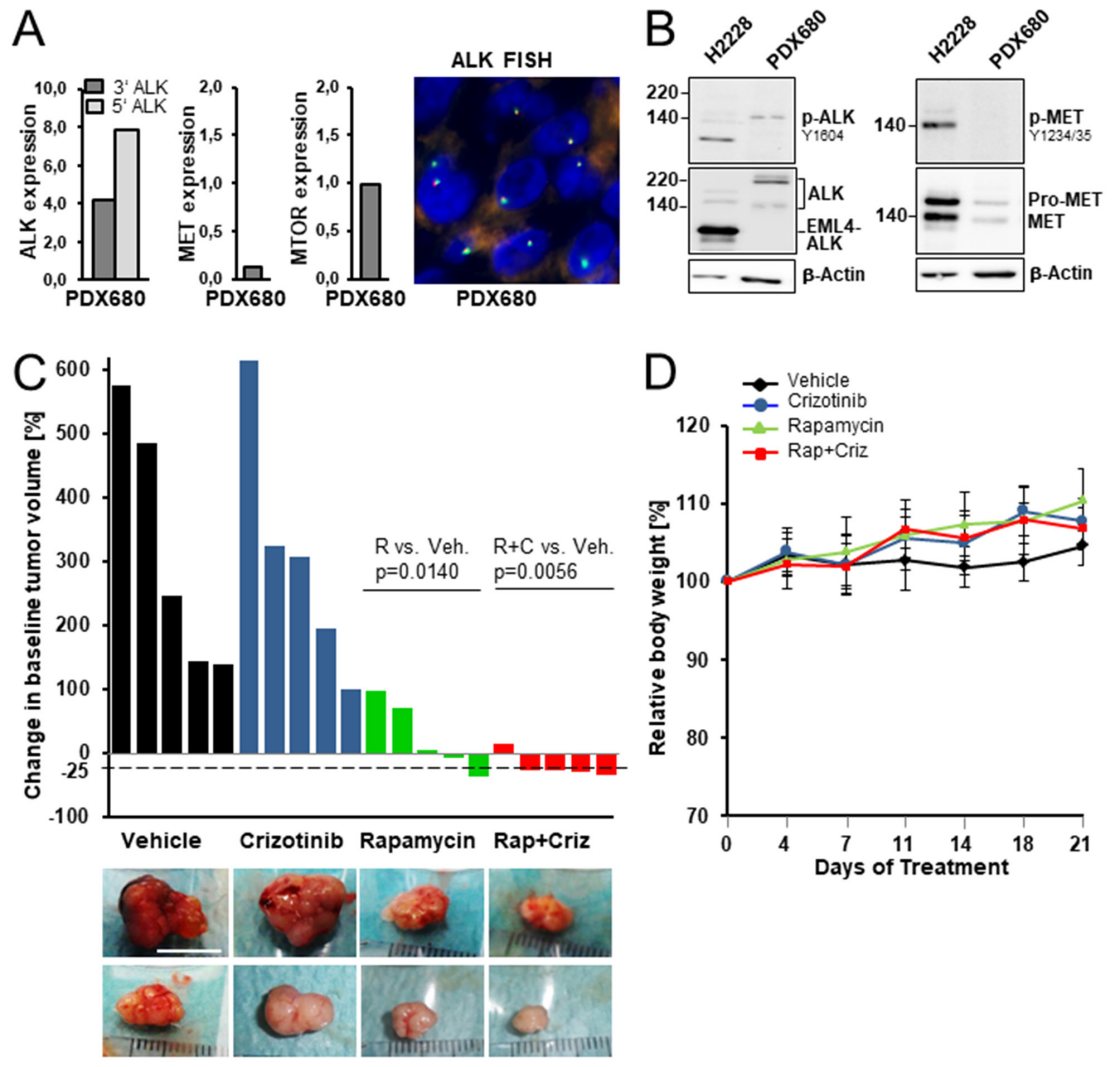
Preclinical efficacy of rapamycin, crizotinib and combined treatment in a mesothelioma xenograft model **(A)** *ALK*, *MET* and *MTOR* transcript expression in xenograft PDX680. *ALK* break-apart FISH confirmed translocation-independent gene expression. **(B)** ALK was activated in PDX680, MET was expressed at low levels, but not phosphorylated demonstrated by immunoblot analysis using cell line H2228 as positive control for active EML4-ALK and MET. **(C)** Waterfall plots of tumoral response in mice treated with vehicle, crizotinib, rapamycin, or rapamycin and crizotinib. Each bar indicated percent change in the volume of an individual tumor on day 21 compared with day 0 of treatment. Statistically significant differences: vehicle vs. rapamycin (p=0.0140), vehicle vs. combined treatment (p=0.0056). Representative pictures of excised tumors from each treatment group; each row contains tumors that had almost identical volumes at day 0 of treatment: upper row, 160-200 mm^3^; lower row, 100-120 mm^3^. Scale bar, 10 mm. **(D)** Changes in the mean of body weight over the time course of the experiment.

To explore the anti-tumor efficacy of simultaneous inhibition of mTOR and ALK, we (1) determined the ALK and mTOR status in eight patient-derived mesothelioma grafts and (2) assessed the effect of rapamycin and crizotinib therapy on the growth of one graft (PDX680) with demonstrated co-activation of both mTOR and ALK. PDX680 overexpressed *ALK* transcripts independent of a gene rearrangement and without kinase-domain mutation and thus resembled *ALK*-expressing patient tumors (Figure 3A). ALK was activated in PDX680, evidenced by phosphorylation of ALK-Tyr1604 (Figure 3B). None of six further PDX tumors with protein lysates available was phospho-ALK positive. The other targets of crizotinib were inactive in PDX680: MET was expressed at low levels but not phosphorylated, and *ROS1* was not expressed (Figure 3A, 3B). PDX680 is thus representative for MPM that express ALK without MET activation. This applies to (1) the four patient samples of our cohort that showed ALK expression, but were negative for MET (Table 1), and (2) MPM that express both kinases but do not activate MET. It is possible that the six patient tumors with co-expression of ALK and MET fall into the second group. However, appropriate (frozen) tissue to investigate phospho-MET in those tumors was not available.

As shown in Figure 3C, crizotinib alone did not exert anti-tumor growth activity on PDX680 grafts, but enhanced the anti-tumor effect of rapamycin. Rapamycin was effective in three of five tumors: Tumor shrinkage (35%-decrease in tumor volume) in one, growth stagnation in two cases. Rapamycin/crizotinib co-treatment was active in all five tumors: Tumor shrinkage (26-34%-tumor decrease) in four, growth stagnation in one case. Crizotinib, rapamycin and combined administrations were all well tolerated, as determined by stable body weights throughout the treatment period (Figure 3D).

### Combined therapy suppresses tumor cell proliferation in a mesothelioma xenograft

**Figure 4:**
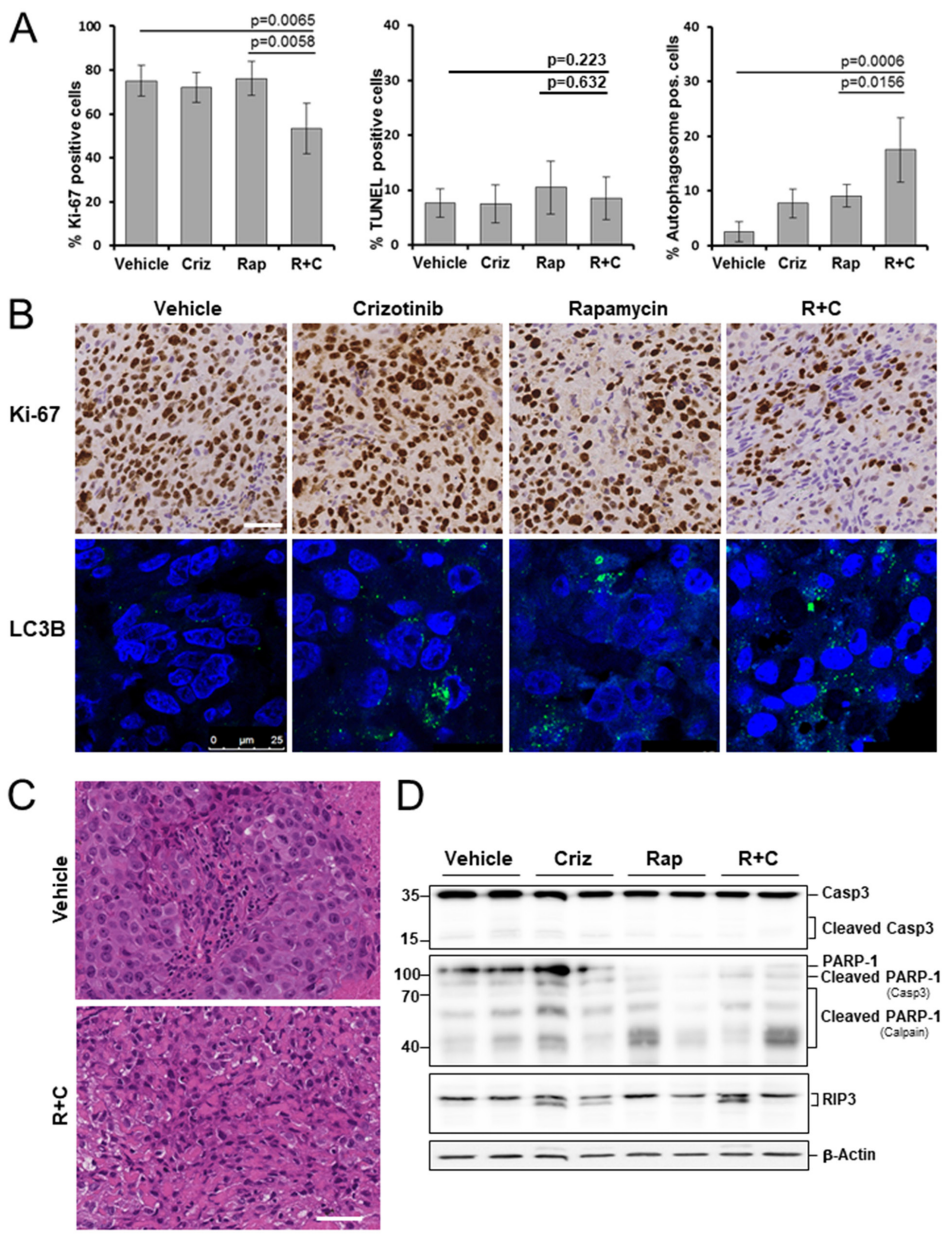
Effects of rapamycin, crizotinib and combined treatment on cell proliferation, apoptosis and autophagy in a mesothelioma xenograft model Mice bearing PDX680 xenografts were treated with vehicle, crizotinib (Criz), rapamycin (Rap), or rapamycin and crizotinib (Rap+Criz) for 21 days. **(A)** Proliferative cells were visualized by immunohistochemical staining with anti-Ki-67, apoptotic cells by TUNEL assay, and autophagy by immunofluorescence monitoring of LC3B incorporation into autophagosomal membranes. Quantification of overall percentage of area positive for Ki-67 (left), TUNEL (middle), LC3B (right). Error bars represent s.d. between individual animals (n=5 per group). **(B)** Representative immunohistochemical images for Ki-67 (top) and immunofluorescence images for LC3B (bottom) of vehicle and treated samples. **(C)** Representative H&E images of tumors treated with vehicle or rapamycin and crizotinib. Note vacuolization, chromatin condensation and loss of cell membrane integrity as effects of combined treatment. **(D)** Immunoblots of PDX680 tumor cells isolated on day 21 after treatment of indicated vehicle or drug (two individual tumors per treatment group). Criz, crizotinib; Rap, rapamycin; R+C, rapamycin and crizotinib; scale bar, 200μm.

We evaluated the anti-proliferative effects of crizotinib and rapamycin in xenografts by Ki-67 immunostaining of histological sections. We did not observe any significant change in the number of Ki-67 positive cells in the crizotinib or rapamycin treated groups, compared to the vehicle control group. Rapamycin/crizotinib co-treatment, however, distinctly decreased the expression of Ki-67 in mesothelioma grafts by 21.8%, compared to the control group (p=0.0065) (Figure 4A, 4B).

### Combined therapy with crizotinib and rapamycin results in caspase-independent cell death

Next, we investigated the effect of different treatments on cell death. Graft tumor samples were examined for apoptosis by assessing DNA fragmentation (TUNEL assay), caspase 3 processing and cleavage of the caspase-3 substrate PARP-1. Surprisingly, treatment with crizotinib, rapamycin, and rapamycin/crizotinib for 21 days produced little apoptosis: 7.5%, 10.5%, and 8.5% of cells, respectively, were positive for the assay, not significantly different from the vehicle control (7.4%) (Figure 4A). Upon all treatments, only slight amounts of active caspase-3 and PARP-1 cleaved by caspase 3 were detected (Figure 4D). These observations indicate that apoptosis was not promoted by treatment. Interestingly, however, rapamycin and rapamycin/crizotinib co-treatment forced proteolytic cleavage of PARP-1 into 40-70-kD fragments, which appear specifically when PARP-1 is digested with calpain in the context of caspase-independent, necrotic cell death (Figure 4D) [27]. Indeed, histomorphological analysis revealed severe features characteristic of necrosis: Co-treatment induced loss of cell membrane integrity, chromatin condensation and vacuolization of the cytoplasm (Figure 4C). Pronounced RIP3 accumulation as a key feature of necroptosis was not observed (Figure 4D). Taken together, rapamycin/crizotinib combination therapy did not promote apoptosis, but induced caspase-independent, necrotic cell death.

### Co-treatment with rapamycin and crizotinib synergistically induces autophagy in MPM cells

It has been reported that mTOR is the main negative regulator of autophagy [18, 28] and that crizotinib induces autophagy in ALK-positive tumors [29–31]. Indeed, we observed autophagy induction by rapamycin and crizotinib in the mTOR/ALK-positive mesothelioma graft model, evidenced by the accumulation of LC3B in autophagosomes: 9.1% and 7.7% autophagosome-positive cells in the rapamycin and crizotinib groups, respectively, versus 2.5% in the control group. There was a significant additive effect when both inhibitors were applied simultaneously: 17.5% (p=0.0156) (Figure 4A, 4B).

### Effect of the co-treatment with rapamycin and crizotinib on downstream signaling pathways

**Figure 5:**
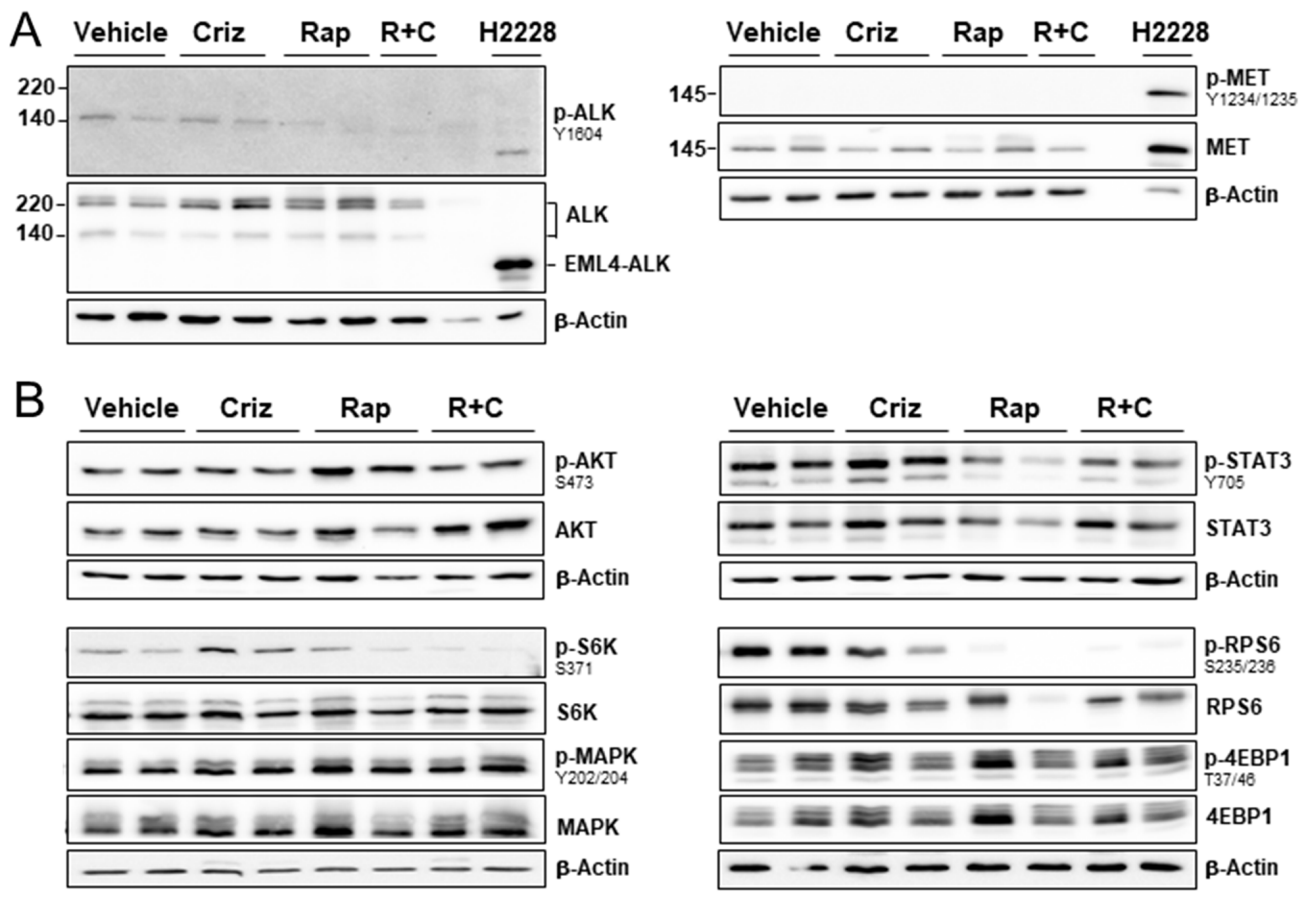
Effect of ALK/MET inhibitor crizotinib and mTOR inhibitor rapamycin on downstream signaling pathways in a mesotheliom xenograft model Immunoblots of PDX680 tumor cells isolated from mice after treatment with vehicle, crizotinib (Criz), rapamycin (Rap), or rapamycin and crizotinib (R+C) for 21 days. **(A)** ALK and MET activity were assessed by using lung cancer cell line H2228 as positive control for the expression of EML4-ALK and active MET. Two tumors each treated with vehicle, crizotinib, rapamycin; one tumor treated with rapamycin/crizotinib. **(B)** Two tumors per treatment group were analyzed for AKT/p-AKT, STAT3/p-STAT3, S6K/p-S6K, and RPS6/p-RPS6 expression.

To gain insight into the molecular mechanisms underlying the effects of rapamycin/crizotinib co-treatment, we evaluated ALK and MET activation in treated graft tumors. Phosphorylation of ALK (140-kD isoform) was not suppressed by crizotinib alone, but when applied in combination with rapamycin, MET was consistently expressed, but only in its non-phosphorylated, inactive form (Figure 5A). In order to clarify the activity of mTOR, we determined the phosphorylation status of the mTOR key substrates ribosomal S6 kinase (S6K), 4EBP1, and AKT. Rapamycin and combination treatment led to a robust dephosphorylation of S6K and its effector RPS6 (Figure 5B). Phospho-4EBP1 was insensitive to treatment in all groups. Rapamycin induced AKT activation, which was suppressed by simultaneous treatment with crizotinib, while crizotinib as a mono-drug did not affect AKT activity (Figure 5B). In addition, co-treatment significantly decreased STAT3 activity, whereas crizotinib alone had no effect (Figure 5B). Phosphorylation levels of MAPK showed no discernible reduction. Taken together, rapamycin/crizotinib co-treatment affected aberrant cell signaling in an mTOR/ALK-positive mesothelioma graft by simultaneous blocking of mTORC1, AKT and STAT3.

## DISCUSSION

In this study, we have investigated the potential of simultaneous therapy of MPM with rapamycin and the ALK/ROS1/MET inhibitor crizotinib. Application of qRT-PCR and IHC analysis on a large cohort of MPM patient samples led to the detection of co-overexpression of mTOR and ALK or mTOR and MET in MPM subgroups of 5% and 15%, respectively. While MET expression had been previously documented [32], balanced full-length *ALK* expression in MPM is described here for the first time. ROS1 was not expressed in our MPM series.

In lung adenocarcinoma and anaplastic large-cell lymphoma, ALK is activated by the expression of chimeric proteins containing the ALK kinase domain, whereas *ALK* activation in neuroblastoma (NB) and rhabdomyosarcoma (RMS) results in the overexpression of wildtype or mutated transcripts [6]. Upregulated expression of non-rearranged transcripts was observed in 25 MPM (19.5%), of which ten expressed ALK protein. Resembling primary MPM, the patient-derived xenograft used in this study expressed non-rearranged *ALK* transcripts and ALK protein in its activated (Tyr1604-phosphorylated) form. Upregulated expression in our MPM cohort was independent of a copy number increase and no activating mutations were seen, while *ALK* activation in NB and RMS is often associated with copy number gains (NB [33], RMS [34, 35]) and kinase-domain mutations (NB [6]). Of interest, recent studies of NB tumor cells have demonstrated that (1) microRNAs (miR-96, miR-424-5p, miR-503-5p) were involved in regulating *ALK* expression [36, 37], and (2) intragenic non-CpG methylation was inversely associated with the amount of *ALK* transcripts [38]. Similarly, ALK overexpression in MPM might be mediated by microRNAs and/or demethylation of intragenic non-CpG sites. *ALK*-wildtype overexpression in NB [39, 40] and RMS [34, 35] primary tumors was associated with poor clinical outcome. In our MPM cohort, ALK expression did not correlate with clinicopathological parameters such as histological growth pattern, asbestos exposition, metastasis, and overall survival. In contrast to our observations, Varesano et al. [41] did not detect ALK protein expression in 63 malignant mesotheliomas by IHC using the antibody ALK1, which however is not as sensitive as the D5F3 antibody employed in our study. Recently, Salvi et al. [42] performed a FISH analysis of crizotinib target genes and found that none of 106 malignant mesotheliomas showed ALK gene rearrangement, which is in line with our finding that *ALK* overexpression was not caused by ALK gene rearrangement (proven by FISH).

Specific ALK inhibitors were shown to inhibit the growth of NB and RMS cells overexpressing wildtype or mutated ALK *in vitro* and in xenograft models [30, 33, 39, 43, 44], and crizotinib is currently being tested for the treatment of NB and RMS (NCT00939770, NCT01524926, NCT01121588). Therefore, it is tempting to speculate that upregulated ALK expression in mesothelioma might be of therapeutic relevance. In this study, crizotinib as mono-drug did not exert anti-tumor efficacy in an ALK-positive xenograft tumor, but was effective when applied simultaneously with rapamycin. In general, failure to achieve tumor growth inhibition by crizotinib could be due to mutations in the ALK kinase domain that effect inhibitor binding or increase ALK catalytic activity or ALK gene amplification both observed in therapy-resistant lung cancer [45]. However, neither ALK gene amplification nor mutation was detected in the graft tumor. Resistance could also be mediated by activation of ALK-independent pathways that hamper the effectiveness of crizotinib, including IGFR, RAS/SRC, EGFR and AKT/mTOR signaling [6, 46]. Indeed, we observed activated AKT/mTOR signaling in the graft tumor (indicated by phosphorylated AKT, S6K, RPS6). mTORC1 inhibition by rapamycin sensitized the tumor to crizotinib: Simultaneous treatment inhibited the activity of ALK and consequently its targets STAT3 and AKT, while crizotinib as single agent did not. In exactly the same way, rapamycin sensitized crizotinib-resistant EML4-ALK+ lung cancer cells to ALK inhibition: Crizotinib alone did not affect ALK and AKT activity, but blocked the feedback activation from mTOR to AKT, when administered together with rapamycin [47]. In those cells, the acquired crizotinib resistance was due to ligand-dependent EGFR activation, which was abrogated by targeting of mTOR by rapamycin (evidenced by decreased EGFR dephosphorylation). It has been reported that aberrant mTOR signaling increases the expression of various growth factors including RTK ligands [48]. Based on those observations, we hypothesize that in our ALK/mTOR positive MPM graft activated mTOR induced ligand-dependent bypass signaling leading to crizotinib resistance. Hence, it seems highly probable that mTOR inactivation sensitized the tumor to crizotinib by suppressing mTOR triggered bypass signaling. The resulting ALK inactivation was able to block the feedback activation from mTOR to AKT (see below).

The PI3K/AKT/mTOR pathway plays an important role in the genesis of various tumors, thus constituting a possible therapy target. Inhibition of mTOR by rapamycin and its derivatives, however, is complicated by adverse AKT activation triggered by release of the negative feedback from mTOR to PI3K. Indeed, increased AKT signaling has been observed in biopsies of cancer patients following everolimus treatment and may explain why rapamycin derivatives have largely cytostatic, but no cytotoxic effects on tumors [49]. Recently, it was shown that the combination of rapamycin or PI3K/mTOR inhibitors and one specific RTK inhibitor (EGFR, MET or IGF1R inhibitor) suppressed adverse AKT activation and hampered mesothelioma cell growth *in vitro* and in MPM xenografts [7, 10, 50].

Here, we have explored the anti-tumor effects of a simultaneous inhibition of mTOR and ALK on the growth of a patient-derived mesothelioma graft with co-activated mTOR and ALK. Our results show that rapamycin alone suppressed tumor growth, resulting in growth stagnation or tumor shrinkage in three of five tumors altogether. In line with other studies [51, 52], rapamycin decreased phosphorylation of the mTOR key substrate S6K and of RPS6, a critical effector of S6K regulated protein synthesis and proliferation. The phosphorylation of the mTORC1 effector 4EBP was insensitive to rapamycin - as described earlier for a variety of cell types [53–55]. While others report on G1 cell cycle arrest and proliferation inhibition induced by rapamycin [52], we did not observe decreased Ki-67 indices. However, we cannot exclude cell cycle arrest, which is not detectable by means of Ki-67 staining. As expected, AKT phosphorylation was enhanced by rapamycin.

Simultaneous treatment of the mTOR/ALK-positive graft with rapamycin and crizotinib was more effective than rapamycin as mono-drug: tumor shrinkage in four and growth stagnation in one of five MPM tumors was achieved. This result is the first of its kind for MPM: similar approaches used in recent studies successfully suppressed tumor growth, but could not induce significant MPM tumor shrinkage [10, 50]. Rapamycin/crizotinib co-treatment was well tolerated, which is in line with recent pre-clinical studies in MPM [50] and other cancers [56] when crizotinib and PI3K/mTOR inhibitors were applied.

Taken together, the combination of rapamycin with crizotinib had three major molecular effects: (1) rapamycin suppressed mTORC1 mediated signaling (indicated by dephosphorylation of S6K and RPS6); (2) rapamycin sensitized the tumor cells to crizotinib, leading to inactivation of ALK signaling (dephosphorylation of ALK, AKT and STAT3); (3) crizotinib blocked AKT feedback activation induced by rapamycin. Simultaneous blocking of mTORC1, STAT3 and AKT might be the underlying molecular mechanism for proliferation inhibition, induction of autophagy and caspase-independent cell death all observed in co-treated tumors, which in concert resulted in tumor shrinkage.

In our study, neither crizotinib nor rapamycin, nor their combination promoted apoptosis in MPM graft tumors. Instead, rapamycin and rapamycin/crizotinib co-treatment induced caspase-independent, necrotic cell death, indicated by (1) PARP-1 cleavage into 40-70-kD fragments, which appears specifically when PARP-1 is digested with calpain in the context of necrosis [27], and (2) histomorphological features characteristic of necrosis: loss of cell membrane integrity, chromatin condensation and vacuolization of the cytoplasm. In keeping with our data, Alvero et al. [57] observed induction of caspase-independent cell death by the mTOR inhibitor NV-128 through inhibition of mTOR and down-regulation of phosphorylated AKT and S6K.

Autophagy represents a pro-survival or pro-death response to several stresses, especially chemotherapy, radiotherapy and starvation, in tumor cells. Under moderate stress conditions, autophagy supplies cells with metabolic substrates, contributing to cell survival, tumor progression and therapeutic resistance. Excessive autophagy, however, is a cell death mechanism that can occur either in the absence of apoptosis or concomitantly with apoptosis [58]. Autophagy has a close relationship with the PI3K/AKT/mTOR pathway [18, 28]. Consistent with the observations made by others that specific PI3K/mTOR inhibitors induce autophagy in MPM cells [59], we also observed autophagy induction when mTOR/ALK-positive MPM tumors were treated with rapamycin. Simultaneous application of crizotinib could effectively block the activity of AKT and significantly enhanced autophagy. It is likely that extensive autophagy contributed to the necrotic cell death observed in tumors co-treated with rapamycin/crizotinib.

In conclusion, we have identified a subgroup of MPM characterized by overexpression of the tyrosine kinase ALK. Because ALK has been successfully targeted with specific inhibitors in other malignancies it is tempting to speculate that activated ALK in MPM might represent a therapeutic target. Indeed, our preclinical data presented here show that combination of the ALK inhibitor crizotinib and the mTOR inhibitor rapamycin results in rapid and robust abrogation of MPM tumor growth *in vivo*. Together, these results suggest that co-treatment with crizotinib and rapamycin should be further explored as a targeted therapeutic alternative in mesothelioma.

## MATERIALS AND METHODS

### Specimen collection

Formalin-fixed paraffin-embedded (FFPE) resection specimens from 144 patients diagnosed with MPM and five normal pleura specimens were included in this study. Triplicate 0.6 mm tumor cores had been inserted into tissue microarrays (TMA) used for all IHC analyses. Lung cancer cell line H2228 (CRL-5935, ATCC, Rockville, MD) served as positive control for *EML4-ALK* and *MET* expression; rhabdomyosarcoma cell line RH30 (DSMZ, Braunschweig, Germany) for the expression of non-rearranged *ALK* transcripts.

### Antibodies and reagents

Antibodies to detect MET (D1C2), mTOR (7C10), LC3B, PARP, Caspase 3, Cyclin D1, phospho-ALK (Tyr1604), phospho-MET (Tyr1234/1235), AKT, phospho-AKT (Ser473), phospho-MAPK (Tyr202/204), 4EBP1, phospho-4EBP1 (Thr37/46), S6K, phospho-S6K (Ser371), RPS6, phospho-RPS6 (Ser235/236), STAT3, phospho-STAT3 (Tyr705) were from Cell Signaling (Danvers, MA, USA). Anti-RIP3 was from Santa Cruz Biotechnology (Dallas, TX, USA), Anti-Ki-67 from Cell Marque (Rocklin, CA, USA), and Anti-β-Actin from Sigma-Aldrich (München, Germany). ALK-1A4 (immunoblotting) was from Origene (Rockville, MD, USA), ALK-D5F3 (immunohistochemistry) was from Ventana Medical Systems (Tucson, AZ, USA). Crizotinib and rapamycin were purchased from Pfizer (Berlin, Germany) and ChemieTek (Indianapolis, IN, USA), respectively.

### Quantitative reverse transcription PCR

Unbalanced *ALK* and *ROS1* transcript expression indicative of ALK/ROS1 gene rearrangement and upregulated gene expression were measured by qRT-PCR amplification of the 5′ and 3′ portions of both genes as described [24, 25]. *MET* and *MTOR* expression was examined using one primer set each (Supplementary Table 2), and qRT-PCR was carried out as described [24]. Tumor expression data were normalized to *PGK1* and compared to the mean value of all pleura samples; the cut-off values for significantly elevated *MET* and *mTOR* expression were 0.55 and 0.14, respectively. *ALK* expression values normalized to *PGK1* were calculated relative to the average value in cell line RH30, which was arbitrarily defined as 1; the cut-off value for relevant *ALK* expression was 0.3.

### PCR and DNA sequence analysis

The *ALK* kinase domain (exons 20-29) was searched for mutations by direct sequencing of PCR products amplified from genomic DNA as described using standard PCR conditions and cycle sequencing [24].

### Fluorescence *in situ* hybridization

FISH was performed as described applying a break-apart probe specific for the *ALK* locus (ZytoLight SPEC ALK Dual Color Break Apart Probe; ZytoVision, Bremerhaven, Germany) [24].

### Immunohistochemistry

IHC was accomplished on 3-μM tissue TMA or full tissue sections. For ALK expression analysis, the ALK D5F3 assay from Ventana was applied. MET and mTOR expressions were assessed with conventional DAB staining on a semi-automated autostainer. After heat-induced epitope retrieval at pH6, anti-mTOR (1:100) or anti-MET (1:100) were incubated for 30 minutes. ALK staining was classified according to the manufacturer’s instructions. MET and mTOR expressions were assessed by estimating the intensity of cytoplasmic and membranous staining: 0/negative, no staining; 1+, faint; 2+, moderate; 3+, strong staining intensity in at least 10% tumor cells.

### Antitumor efficacy study

Eight MPM patient-derived xenograft models were established by Charles River Discovery Research Services (Freiburg, Germany). Tumor cells were implanted subcutaneously in NMRI nu/nu mice and allowed to grow to reach a tumor size of 50-200 mm^3^. Tumor-bearing mice were randomized into different treatment groups (n=5/group): Vehicle control (orally), crizotinib (100 mg/kg/d, orally), rapamycin (7.5 mg/kg/2d, i.p.), or combination treatment with crizotinib (100 mg/kg/d, orally) and rapamycin (7.5 mg/kg/2d, i.p.). The length (L) and width (W) of each tumor was measured with calipers, and the body weight was determined every 3 to 4 days. The tumor volumes were TV=L*W^2^*0.5; the relative tumor volumes of individual tumors were RTV=TV_21_/TV_0_*100. After 21 days of treatment, mice were sacrificed, solid tumors were excised and parts either processed for paraffin embedding or fresh-frozen for protein analysis.

### Quantification of apoptosis

The TUNEL assay was accomplished on 3-μm FFPE sections employing the ApopTag-Plus Kit (Merck-Millipore, Darmstadt, Germany). After pretreatment (20 mg/ml proteinase) and quenching (3% H_2_O_2_) sections were treated with TdT enzyme and anti-digoxigenin-peroxidase, followed by DAB detection. Negative controls were incubated in medium lacking TdT enzyme. Immunoreactivity in nuclei and apoptotic bodies was considered positive. The apoptosis index corresponded to TUNEL-labelled cells among at least 900 cells per tumor and was expressed as the percentage of positive cells.

### Analysis of necroptosis

Immunoblot analysis with anti-RIP3 (1:500) and anti-PARP (1:1000) was used to investigate cleavage products of RIP3 and PARP that are indicative of necroptotic cell death. Immunoblotting was performed as described below.

### Quantification of proliferation

3-μm FFPE tissue sections were stained with Ki-67 antibody using conventional DAB staining on a semi-automated autostainer. After heat-induced epitope retrieval at pH9, anti-Ki-67 (1:100) was incubated for 30 minutes. Negative controls were obtained by omitting the primary antibody. Only nuclear Ki-67 immunoreactivity was considered positive. The proliferation index corresponded to the number of labelled Ki-67 cells among at least 670 cells per tumor and is expressed as percentage.

### Quantification of autophagy

LC3B incorporation into autophagosomal membranes was monitored by immunofluorescence staining of 3-μM FFPE tissue sections. After heat-induced epitope retrieval at pH6, cells were incubated with anti-LC3B (1:200) overnight at 4°C and Alexa Fluor 488-conjugated anti-rabbit IgG (1:250; Thermo Fisher Scientific) for 1 h at room temperature. DAPI was used for nuclear staining. Images were taken on a confocal microscope Leica TCS SP8. The number of LC3B positive dots was quantified using ImageJ software (NIH, Bethesda, MD, USA), cells with more than five dots were considered positive. The autophagy index corresponded to the number of autophagosome positive cells among at least 580 cells per tumor and was expressed as percentage.

### Gel electrophoresis and immunoblotting

Total protein extracts were prepared by lysing frozen PDX tumor cells in FastPrep lysing matrix tubes (MP Biomedicals, Illkirch, France) with lysis buffer containing cOmplete protease inhibitor cocktail, PhosSTOP phosphatase inhibitor (Roche, Mannheim, Germany). 30 μg protein were separated by SDS-PAGE and transferred onto nitrocellulose membranes. For immunoblotting, membranes were probed with primary antibodies overnight at 4°C, then incubated with anti-rabbit IgG-HRP (Cell Signaling). Signals were detected by enhanced chemiluminescence (SuperSignal WestDuraExtendedDurationSubstrate, Thermo Fisher Scientific) using CCD camera STELLA3200 (Raytest, Straubenhardt, Germany). Antibodies were used at a dilution of 1:1000, except anti-β-actin (loading control; 1:10000).

### Statistical analysis

Differences between groups were assessed by two-tailed student’s *t*-test (continuous data) or Fisher’s exact test (categorial data). A *P* value less than 0.05 was considered significant. For all calculations, Analyse-it software (Leeds, United Kingdom) for Microsoft Excel was used.

### Ethics statement

The investigations were conducted according to the ethical standards of the Declaration of Helsinki and according to national and international guidelines. This study was approved by the Ethics Committee of the University Hospital, Tübingen, Germany (344/2012BO2). Animal work was approved by the Regional Administrative Authority of Freiburg, Germany (G-13/13).

## SUPPLEMENTARY MATERIALS TABLES


