# Supplementary material for: A subgroup of pleural mesothelioma expresses ALK protein and may be targetable by combined rapamycin and crizotinib therapy
Source: Oncotarget. 2018 Apr 17;9(29):20781–94. doi: 10.18632/oncotarget.25111 (PMC5945506; doi:10.18632/oncotarget.25111)
Supplement: Supplementary file 1 [file oncotarget-09-20781-s001.pdf]

A subgroup of pleural mesothelioma expresses ALK protein and may be targetable by combined rapamycin and crizotinib therapy

SUPPLEMENTARY MATERIALS

Supplementary Table 1: Correlation between mTOR, ALK and MET protein expression and clinicopathological parameters

Clinicopathological characteristics		mTOR			ALK			MET		
		positive (n=14)	negative (n=32)	P	positive (n=9)	negative (n=41)	P	positive (n=19)	negative (n=27)	P
Age (years)	Mean	71.9	69	0.3335	67.9	70.1	0.5120	67	71.9	0.0674
	Range	54-89	51-86		51-86	54-89		51-80	58-89	
Sex	Male:female	12:2	27:5	1.0000	7:2	35:6	0.6231	15:4	24:3	0.4244
Asbestos exposition	Yes:no	12:2	27:5	1.0000	6:3	36:5	0.1437	15:4	24:3	0.4244
Metastasis at presentation	Yes:no	3:11	10:22	0.7241	1:8	15:26	0.2396	6:13	7:20	0.7463
Histological subtype	E:S:B[a]	12:0:2	22:4:6	0.3261	7:0:2	31:4:6	0.5632	17:0:2	17:4:6	0.0929
Overall survival (months)	Mean	23.5	22.4	0.8983	19.2	23.8	0.6411	23.9	22	0.8236
	Range	2-60	3-145		2-40	3-145		4-111	2-145	

[a]Histological growth pattern: E, epithelioid; S, sarcomatoid; B, biphasic.

Supplementary Table 2: Oligonucleotide primers for quantitative RT-PCR amplifications

Primer	Gene	Oligonucleotide sequence[a]
ALK_TM3	*ALK* (ex.25)	TCAAGAGGCAGTTTCTGG
ALK_TM4	*ALK* (ex.24)	TCGGGACATTGCCTGTGG
ALK_TM5	*ALK* (ex.4/5)	AGGGAAGCATGGTTGGAC
ALK_TM6	*ALK* (ex.5)	GCCACTCGAAATGGGTTG
ROS1_TM1	*ROS1* (ex.15)	AAGACATATGTGATACAAAGGC
ROS1_TM3	*ROS1* (ex.16)	AATAGCTTCACGTGGGTAAC
ROS1_TM22	*ROS1* (ex. 17)	CTCCTGTATTGGTTGGTTC
ROS1_TM23	*ROS1* (ex.17/18)	TATCCCCAGTGCTCTGTC
ROS1_TM4	*ROS1* (ex.40)	ACTACTCAATCTGATGTATGG
ROS1_TM5	*ROS1* (ex.41)	ATGAGCTGGATAAGGCTG
MET_TM2	*MET* (ex.20)	AACCCGAATACTGCCCAG
MET_TM3	*MET* (ex.21)	GGCTTTAGGGTGCCAGC
MTOR_TM9	*MTOR* (ex.28)	CTACACTACAAAGAACTGGAG
MTOR_TM12	*MTOR* (ex.29)	GCTGTAGCTTATTATTAATGCTG
PGK1_TM_C	*PGK1* (ex. 8)	CTGCTGGCTGGATGGGC
PGK1_TM_D	*PGK1* (ex. 9)	ATCTGCTTAGCCCGAGTG

[a]Oligonucleotides were designed based on published sequence data (NM_002944; NG_033929; NM_004958; NM_000245).
